# Advancing targeted therapy for colorectal cancer: harnessing ligand-directed enzyme prodrug therapy for highly specific interventions

**DOI:** 10.3389/fonc.2025.1570712

**Published:** 2025-05-29

**Authors:** Hend Al-Jaber, Kabir H. Biswas, Layla Al-Mansoori

**Affiliations:** ^1^ College of Health & Life Sciences, Hamad Bin Khalifa University, Doha, Qatar; ^2^ Biomedical Research Center (BRC), Qatar University, Doha, Qatar; ^3^ Department of Biomedical Sciences, College of Health Sciences, Qatar University, Doha, Qatar

**Keywords:** colorectal cancer, targeted therapy, prodrug, drug resistance, carboxypeptidase G2, fusion protein

## Abstract

Colorectal cancer (CRC) is a significant global health burden, ranking as the third most frequently diagnosed cancer and a leading cause of cancer-related mortality. Current therapeutic modalities face challenges in advanced stages, including drug resistance, toxicity, and off-target effects. Ligand-Directed Enzyme Prodrug Therapy (LDEPT) has emerged as a promising strategy to address these limitations by delivering cytotoxic agents directly to tumor sites, minimizing damage to healthy tissues. LDEPT employs ligand-enzyme complexes that specifically target cancer cells, where the enzyme activates a prodrug into its cytotoxic form, enhancing precision, reducing adverse effects, and improving the therapeutic index compared to conventional chemotherapy. This review provides a detailed analysis of LDEPT’s core components while highlighting recent advancements in the field. Preclinical studies demonstrate promising outcomes, and initial clinical trials validate its potential. However, challenges remain, including optimizing ligand specificity, improving stability and delivery of ligand-enzyme complexes, and mitigating immune responses that may compromise effectiveness. Integrating LDEPT with immunotherapies or conventional chemotherapies could yield synergistic effects, paving the way for more comprehensive and personalized CRC treatment strategies. Continued research and clinical validation are essential to refine these approaches and transition LDEPT from experimental studies to routine clinical practice, with the potential to transform the treatment paradigm for advanced CRC.

## 1 Introduction

Colorectal cancer (CRC) remains a significant global health concern. According to the International Agency for Research on Cancer (IARC), it is the third most commonly diagnosed cancer worldwide, with approximately 1.85 million new cases annually (1, 2). There were approximately 1.85 million new cases of CRC globally in 2020, contributing to 9.4% of all cancer cases. The burden of CRC is particularly high in high-income countries, though increasing incidence rates are being observed in low- and middle-income countries as well. In the United States, CRC is the second leading cause of cancer-related mortality, with projections for 2025 estimating approximately 154,270 new cases—107,320 cases of colon cancer (54,510 in men and 52,810 in women) and 46,950 cases of rectal cancer (27,950 in men and 19,000 in women) (3). Although early-stage CRC is often curable, high recurrence rates and the emergence of drug resistance continue to hinder long-term treatment success (4). The disease remains highly prevalent in both sexes. While overall incidence rates declined by about 1% annually from 2012 to 2021—primarily due to increased screening and lifestyle changes—rates among individuals under 50 rose by 2.4% each year during the same period (3). Despite advancements in screening and treatment, disparities in healthcare access continue to contribute to unequal outcomes globally. Moreover, despite significant progress in treating other types of cancer, CRC management still falls short of achieving satisfactory outcomes (4). The global incidence of CRC is projected to rise substantially by 2030, highlighting the urgent need for more effective prevention and treatment strategies (5).

In Qatar, CRC represents a significant public health issue, being the second most commonly diagnosed malignancy in men and the third in women (6). A substantial portion of CRC cases in Qatar are diagnosed at advanced stages, hindering early detection and timely intervention. This delayed diagnosis can be attributed to several factors, including limited public awareness, insufficient participation in screening programs, and the asymptomatic nature of the disease in its early stages. To mitigate these challenges, targeted awareness campaigns and improved access to screening services are crucial. Additionally, understanding the unique genetic and environmental factors influencing CRC in the Qatari population can inform the development of more tailored prevention and treatment strategies.

Colorectal cancer is frequently asymptomatic in its early stages, with clinical manifestations typically emerging in advanced stages (III and IV). These symptoms may include abdominal pain, alterations in bowel habits such as diarrhea or constipation, cramping, unintentional weight loss despite preserved appetite, rectal bleeding, as well as systemic signs of disease such as muscle weakness and fatigue (7). Unfortunately, the often-insidious nature of the disease, with early-stage symptoms being subtle or absent, frequently leads to the late-stage diagnoses, limiting the efficacy of curative surgical interventions.

Standard treatment modalities for CRC typically include a combination of surgical resection, chemotherapy, and radiation therapy, tailored to the tumor’s stage and anatomical location. In advanced cases, targeted therapies and immunotherapies are increasingly utilized to improve patient outcomes and control disease progression. However, despite these interventions, the five-year survival rate for CRC remains around 64%, with a marked decline to 12% in patients with metastatic disease, highlighting the critical need for more efficacious therapeutic options (6, 8).

For patients with unresectable lesions or those ineligible for surgical intervention, treatment priorities shift toward achieving maximal tumor reduction and controlling disease progression. In these cases, radiotherapy and chemotherapy serve as the primary therapeutic modalities. Additionally, chemotherapy or radiotherapy may be employed as neoadjuvant or adjuvant therapies to shrink or stabilize the tumor before or after surgical resection (9). In metastatic colorectal cancer (mCRC), which affects approximately 50-60% of CRC patients, standard treatment consists of antineoplastic agents aimed at prolonging survival and enhancing quality of life (10).

While significant strides have been made in CRC treatment, challenges persist, particularly for patients with advanced or metastatic disease. Despite advancements in targeted therapies, immunotherapies, and surgical techniques, the high incidence of late-stage diagnoses and the complex nature of metastatic disease continue to hinder optimal outcomes. To address these challenges, innovative approaches such as Ligand-Directed Enzyme Prodrug Therapy (LDEPT) offer promising solutions. By precisely delivering cytotoxic agents to tumor cells, LDEPT aims to maximize therapeutic efficacy while minimizing systemic toxicity. This targeted approach has the potential to revolutionize CRC treatment, especially for patients with advanced disease or those who have developed resistance to conventional therapies. Continued research and clinical validation are crucial to fully realize the potential of LDEPT and improve patient outcomes worldwide.

This review focuses on exploring innovative therapeutic strategies, particularly Ligand-Directed Enzyme Prodrug Therapy (LDEPT), as a promising approach for the treatment of advanced and metastatic colorectal cancer. By addressing the limitations of conventional therapies and emphasizing the potential of targeted treatment modalities, this review aims to provide a comprehensive understanding of LDEPT’s mechanisms, clinical applications, and future prospects. Given the global burden of CRC and the persistent challenges in managing late-stage and resistant cases, this discussion is both timely and critical. Advancing knowledge in this area could pave the way for more effective and personalized interventions, ultimately improving patient outcomes and reducing the global impact of CRC.

## 2 Comprehensive insights into colorectal cancer management

Effective management of CRC requires a multifaceted approach encompassing prevention, early detection, and tailored treatment strategies. Routine screening and risk assessment are cornerstone practices in reducing CRC incidence and mortality, particularly by identifying individuals at an elevated risk for developing the disease (8).

Routine screening of individuals at average risk is the most effective strategy for preventing CRC and reducing CRC-related mortality in the general population (11). However, certain individuals face a higher risk due to genetic, personal, and lifestyle factors. Age is a significant risk factor, with most cases occurring in people over the age of 50 (12). A family history of colorectal cancer, particularly if a first-degree relative was diagnosed before age 50, substantially increases risk, as does a personal history of colorectal polyps or previous cancers, such as ovarian, uterine, or breast cancer (13). Inherited genetic conditions, such as Lynch syndrome and familial adenomatous polyposis (FAP), are linked to a notably high risk of CRC, often resulting in early-onset and more aggressive disease forms (14).

Individuals with chronic inflammatory bowel diseases, like ulcerative colitis and Crohn’s disease, are also at an increased risk due to prolonged colon inflammation (15). Lifestyle factors play a significant role; diets high in red and processed meats, low fiber intake, physical inactivity, obesity, smoking, and heavy alcohol consumption have all been associated with an elevated risk of CRC. Furthermore, type 2 diabetes is linked to a higher risk, potentially due to insulin resistance and associated metabolic changes (16). The interplay between these risk factors and the molecular mechanisms of CRC progression underscores the importance of early detection and innovative therapeutic strategies tailored to these pathways. Insulin resistance leads to elevated levels of insulin and insulin-like growth factors, which can promote the growth of cancer cells (17). Additionally, the chronic inflammation and alterations in the gut microbiome commonly seen in individuals with type 2 diabetes may further contribute to CRC development. Dysregulated glucose metabolism and obesity, often present in people with type 2 diabetes, are known to exacerbate the risk of colorectal carcinogenesis, highlighting the complex interplay between metabolic dysfunction and cancer progression (18, 19). Moreover, individuals who have received radiation therapy to the abdomen or pelvis for other cancers may have an increased risk of developing colorectal cancer later in life.

Understanding and identifying these risk factors are crucial for targeted screening and prevention strategies. Consequently, population-based screening programs have been implemented in many regions, including European countries, Canada, select areas of North and South America, and parts of Asia and Oceania (20). These screening initiatives primarily aim to identify asymptomatic cases of CRC among average-risk populations, enabling early-stage interventions that significantly reduce the disease burden on individuals and communities (21).

However, despite these advancements, the progression of CRC remains a slow process, typically requiring 10 to 15 years for a polyp to develop into a malignant tumor. Regular screening, early detection, and removal of polyps are critical for CRC prevention. Current diagnostic methods are capable of detecting only approximately 40% of CRC cases at an early stage, and the risk of recurrence persists following surgical resection and adjuvant therapies. Moreover, chemotherapeutic agents, while targeting cancerous cells, also tend to harm surrounding healthy cells. Additionally, resistance to modern chemotherapeutic agents is commonly acquired by nearly all CRC patients, resulting in reduced efficacy of anticancer treatments and ultimately leading to chemotherapy failure (4).

Colorectal cancer exhibits considerable genetic diversity and can arise through a range of distinct mechanisms. CRC cells often present a high burden of somatic mutations, resulting in varied gene expression profiles. This extensive mutational load makes CRC one of the most mutation-rich cancers identified to date (22). In addition to mutation-based classifications, there has been significant progress in developing a novel categorization system based on gene expression profiles. This system was developed through comprehensive analyses that combined gene expression data with tumor genotyping. These advanced classification frameworks have been refined with emerging data, deepening our understanding of CRC heterogeneity and supporting more personalized treatment approaches (4). By integrating genetic mutation data with expression profiles, this approach enables more precise stratification of CRC subtypes, potentially enhancing diagnostic accuracy and tailoring therapeutic strategies. Researchers aim to continue to gain further insights into CRC pathogenesis and treatment responses, paving the way for innovations in clinical management (22). By identifying specific molecular subtypes, these classification systems can help clinicians select the most appropriate treatment options for individual patients. For example, patients with certain genetic mutations may benefit from targeted therapies that inhibit specific molecular pathways involved in tumor growth and progression.

## 3 The complex landscape of colorectal cancer: from initiation to treatment

Building on the identification of key risk factors, it is essential to delve into the molecular pathways of carcinogenesis, which offer insight into how these factors contribute to CRC development through genetic and epigenetic alterations. The initiation of CRC arises when epithelial cells accumulate a series of genetic or epigenetic alterations that drive their excessive proliferation (23). These proliferating cells initially form a benign adenoma, which can progress to cancer and metastasize through various mechanisms, including microsatellite instability, chromosomal instability, and serrated neoplasia (24, 25). The term “adenoma-carcinoma sequence” describes the typical progression of CRC, with the majority of sporadic cases following this pathway. It begins with the formation of a small adenoma, which enlarges into a larger adenoma and eventually develops into cancer. This pathway is strongly associated with the chromosomal instability positive subtype of CRC and is responsible for approximately 10–15% of sporadic cases. It is characterized by progression from normal cells to hyperplastic polyps, sessile serrated adenomas, and ultimately cancer (26). Another pathway involves the CpG island methylator phenotype (CIMP)-high subtype, often associated with inflammation. In this pathway, chronic inflammation leads to a sequence of changes from normal cells to indeterminate dysplasia, advancing to low-grade dysplasia, then high-grade dysplasia, and finally cancer (26). This inflammatory route is typically implicated in CRC development but accounts for less than 2% of cases worldwide, including those due to inflammatory bowel diseases and the use of prophylactic colectomy. In all pathways, benign precursor lesions can be identified and removed, though they are most prominently observed in the adenoma-carcinoma and serrated pathways. Since these precursor lesions take years to evolve into cancer, there is a critical window for secondary prevention through early detection and intervention (4).

Once an adenocarcinoma becomes invasive, it has the potential to disseminate to other regions of the body through the blood and lymphatic systems. Adenocarcinomas constitute approximately 96% of all CRC (27). The progression from polyp to invasive cancer can span up to 18 years, with metastasis typically taking an average of nine years to develop. CRC, like other cancers, is staged from stage 0 (carcinoma *in situ*) to stage IV (28).

**Figure 1:**
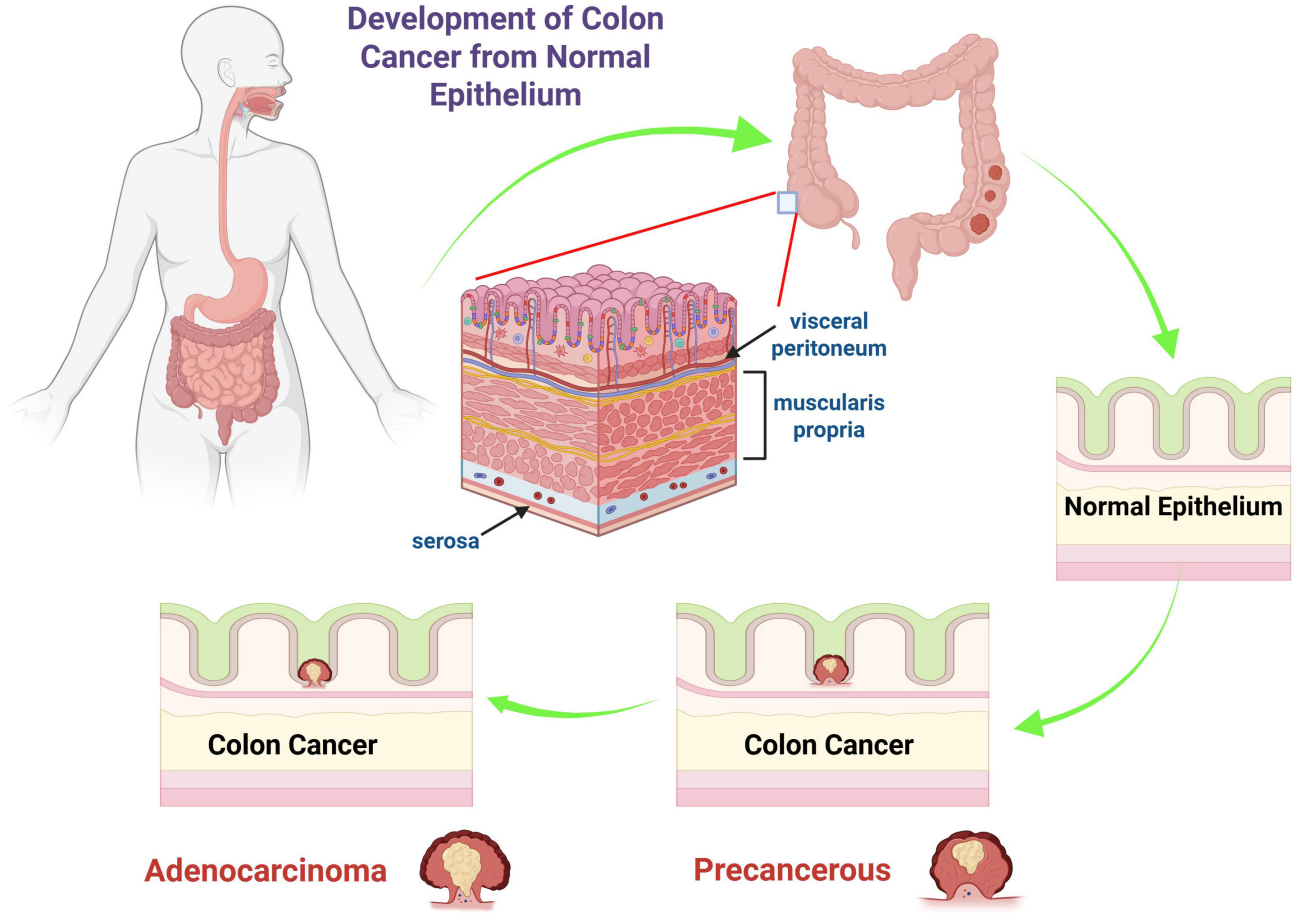
The diagram illustrates the progression of colon cancer from normal epithelium to adenocarcinoma. It shows the anatomy of the colon, highlighting the layers such as the serosa, muscularis propria, and visceral peritoneum. The sequence begins with normal epithelium, progresses to a precancerous state, and ultimately to colon cancer, specifically adenocarcinoma. The arrows depict the stepwise transformation, emphasizing the changes in epithelial cells that lead to malignancy.

During the progression from benign to malignant stages, dysplastic tissue may form a tumor that, after accumulating multiple aberrant DNA alterations, develops into CRC. A benign soft tissue tumor is characterized by its lack of metastasis to other anatomical sites. Hyperproliferation can lead to the development of benign polyps or adenomas (stage 0). Approximately 10% of adenomatous polyps may progress to malignancy, forming adenocarcinomas that invade the muscularis propria (stage I). The tumor then continues to expand and invade the serosa (stage II) and the visceral peritoneum (stage III) (**Figure 1**). At stage IV, there is a potential for lymphatic or hematogenous metastasis (4). The disease stage dictates the severity and informs the therapeutic approach (29). For stages 0–II CRC, surgical resection is the standard treatment. Stage III CRC requires surgical intervention in conjunction with adjuvant chemotherapy. In cases of stage IV and recurrent CRC, treatment typically includes surgery, chemotherapy, and targeted therapy, though a definitive cure has not yet been established (4).

Mutations in genes such as adenomatous polyposis coli (APC), DNA mismatch repair genes, KRas, and p53 lead to dysregulated cell proliferation and the progression to CRC, often aligning with distinct stages of tumorigenesis (30–32). These mutations result in the production of dysfunctional proteins that fail to perform normal cellular regulatory functions, allowing the continued proliferation of cells with damaged DNA and the accumulation of additional genetic alterations, ultimately giving rise to a malignant phenotype (32). The molecular changes associated with these mutations are typically undetectable during the early stages of CRC, complicating early diagnosis and contributing to a high mortality rate. Beyond challenges in early detection, CRC diagnosis faces additional complexities, such as accurate pre-operative staging and the identification of lymph node involvement and micro-metastatic disease through advanced imaging techniques, both of which are crucial for optimizing patient management (4). Understanding these molecular alterations can help identify patients who may benefit from targeted therapies or clinical trials. For example, patients with specific mutations in certain genes may be eligible for treatments that target those particular molecular pathways.

Surgery remains the primary treatment modality for CRC, particularly in its early stages (stage 0 to stage II) (33). In advanced stages, additional interventions such as chemotherapy or targeted therapies are often required. The use of laparoscopic resection for early-stage rectal cancers remains a subject of debate due to concerns about its efficacy. Given the high incidence of CRC, the development and standardization of widely applicable surgical techniques are critical, which also underscores the importance of comprehensive surgical training programs (33). Pathological evaluation of resected tumor specimens plays a crucial role in postoperative care and prognosis. However, challenges in examining and reporting these specimens often arise due to the complexity of applying existing pathological criteria or the introduction of new diagnostic concepts. Accurate and detailed pathology reports are essential for informing prognosis and guiding patient management (34, 35).

Drug resistance presents a significant challenge in the treatment of CRC. Despite advances in chemotherapy, many patients develop resistance to these drugs, reducing their effectiveness and leading to treatment failure. This limitation has prompted a shift in focus towards the development of targeted therapies, which offer a more precise approach to cancer treatment. Unlike conventional chemotherapeutic agents, which can damage both cancerous and healthy cells, targeted therapies specifically attack cancer cells by interfering with molecular pathways critical for tumor growth and survival. By targeting specific genetic and molecular abnormalities unique to cancer cells, these therapies aim to reduce side effects, improve patient outcomes, and overcome drug resistance (36). Colorectal cancer remains a formidable challenge due to its genetic heterogeneity, potential for late-stage diagnosis, and the limitations of existing treatment modalities. While surgery continues to be the cornerstone for early-stage CRC treatment, the emergence of targeted therapies offers a promising avenue for more personalized and effective strategies, particularly in advanced or recurrent cases. Continued advancements in understanding the molecular and genetic basis of CRC will be crucial in developing more effective diagnostic tools and therapeutic options. With ongoing research and innovation, there is hope for improved survival rates and better quality of life for patients affected by this complex disease (37, 38).

## 4 Transforming colorectal cancer care through molecular precision

Targeted therapy and personalized medicine are rapidly evolving fields aimed at improving cancer treatment and prevention strategies, particularly in CRC. One of the most significant breakthroughs in contemporary oncology is the transition from a traditional organ-based approach to a personalized approach guided by detailed molecular analysis. This paradigm shift, emphasizing the specific molecular alterations within a tumor, has facilitated the development of tailored treatment options. By leveraging targeted therapies, researchers and clinicians can now select treatments based on the molecular profile of a patient’s cancer (39). This shift has been particularly transformative in CRC care, where advances in molecular precision are reshaping treatment paradigms.

Recent advancements in targeted therapies have revolutionized CRC treatment. Unlike traditional chemotherapeutic agents, which indiscriminately target rapidly proliferating cells, targeted therapies selectively interfere with molecular pathways critical to tumor growth and survival. By focusing on specific genetic and molecular alterations unique to cancer cells, these therapies aim to minimize off-target toxicity, reduce adverse events, and overcome drug resistance. This precision not only improves patient outcomes but also represents a significant step toward more personalized cancer care (38). The ongoing development of targeted therapies, fueled by a deeper understanding of the molecular and genetic basis of CRC, marks a pivotal advancement in oncology. As research continues to unravel the complexities of CRC pathogenesis, these targeted approaches hold the potential to enhance diagnostic accuracy and refine treatment strategies, ultimately improving survival rates and quality of life for CRC patients (40).

While the concept of molecular targeted therapy originated in the early 1900s, its application in cancer treatment, particularly in CRC, has gained substantial momentum in the last two decades. During this time, the approach has been revitalized, experiencing significant advancements and development (41, 42). Targeted therapies can impact tumor growth and progression through various mechanisms. By directly engaging specific molecular pathways within cancer cells, these therapies can inhibit cellular proliferation, differentiation, and migration. Moreover, targeted therapies have the ability to alter the tumor microenvironment, including local vasculature and immune cell interactions, thereby suppressing tumor growth and enhancing immune surveillance (43, 44).

Key components of targeted therapy include small molecules and monoclonal antibodies, which target specific molecular pathways involved in cancer cell growth and survival. Small molecules, typically with molecular weights below 900 Da, can enter cells and disrupt critical cellular functions by inhibiting specific enzymes. This disruption can impede tumor cell proliferation and even induce programmed cell death. Notable molecular targets for these small molecules include cyclin-dependent kinases, proteasomes, and poly ADP-ribose polymerase. Examples of small molecule targeted therapies include carfilzomib, ribociclib, and rucaparib (42). Monoclonal antibodies, on the other hand, target extracellular components such as cell surface receptors. These antibodies bind to specific antigens, directly influencing downstream signaling pathways that control cell cycle progression and apoptosis. Certain monoclonal antibodies can engage with immune cells, modulating the immune response to enhance the targeted attack on cancer cells (45).

Achieving precise delivery of anti-cancer drugs to solid tumors at effective therapeutic doses remains a significant challenge. A promising approach to address this issue involves utilizing targeting ligands that are selectively recognized and internalized by cancer cells and/or other components of the tumor microenvironment, while minimizing interaction with healthy cells. Various types of ligands—such as proteins, peptides, antibodies, and nanobodies—have been employed in this strategy (46). The identification of novel targeting moieties with improved specificity for tumors is essential for advancing targeting strategies that reduce interactions with normal tissues. Enhanced specificity can significantly mitigate off-target effects and improve the precision of targeted therapies. Addressing the complexity of the tumor microenvironment underscores the critical need for the development of novel ligands with multi-targeting capabilities, which could significantly enhance therapeutic efficacy and overcome the limitations of current treatment modalities (46).

While targeted therapies offer significant promise, effective drug delivery to solid tumors remains a major challenge. Ligand-targeted therapeutics (LTT) are emerging as a promising approach to overcome this challenge. With the ongoing evolution of targeted therapies, LTTs have emerged as a key focus, particularly in addressing the complex molecular landscape of CRC.

## 5 The evolving landscape of ligand-targeted therapeutics in CRC

Ligand-targeted therapies (LTTs) are emerging as a promising strategy to revolutionize CRC treatment by selectively targeting tumor cells while sparing healthy tissues. Several forms, including radioimmunotherapies, immunotoxins, and immunoconjugates, have already received clinical approval, while many others are currently in clinical trials. Advances in antibody engineering, including the development of humanized or fully human antibody fragments and the use of phage-display techniques, have enabled the identification of new high-affinity targeting moieties (47). Key factors influencing the success of LTTs include receptor expression levels, ligand internalization, choice of targeting agents (such as antibodies or non-antibody ligands), and ligand binding affinity. Innovative approaches, such as crosslinked antibody fragments, bispecific antibodies, and fusion proteins, are being explored to enhance therapeutic efficacy. LTT principles also extend to microreservoir systems like liposomes and polymers, which can deliver higher therapeutic payloads per targeting molecule and provide sustained drug release (47, 48). Ongoing research is needed to optimize drug-release rates, pharmacokinetics, biodistribution, and to understand mechanisms underlying certain side effects. Clinical testing strategies and intellectual property considerations also remain important challenges. Additionally, LTTs can be applied to the targeted delivery of gene-based therapies, such as antisense oligonucleotides (47). By addressing the limitations of traditional therapies and harnessing the power of targeted delivery, LTTs hold the potential to significantly improve patient outcomes and quality of life.

## 6 Challenges in conventional chemotherapy and emerging strategies for targeted drug delivery

Despite advances CRC treatment, significant gaps in efficacy remain, particularly for patients diagnosed at advanced stages. While surgery offers curative potential for early-stage disease (49), the majority of patients present with metastatic or advanced CRC, where the five-year overall survival rate is approximately 13% (50). Systemic chemotherapy, the mainstay for these patients, offers limited median overall survival (mOS) of 17-23 months (51, 52). The prognosis for metastatic CRC (mCRC) has improved with targeted therapies, including antibodies against epidermal growth factor receptor (EGFR) and vascular endothelial growth factor (VEGF), as well as tyrosine kinase inhibitors (TKIs) (53, 54). However, the benefits of these therapies are often constrained by resistance, side effects, and variability in patient responses.

Effective drug delivery is crucial for improving cancer therapies; however, conventional chemotherapy struggles with the precise targeting of therapeutic agents. Targeted therapies are hampered by nonspecific delivery, leading to severe off-target side effects, inadequate distribution to target organs, and rapid clearance, which ultimately reduce their therapeutic efficacy and compromise the therapeutic index. Moreover, the lack of tumor specificity in conventional approaches contributes to drug resistance, as cancer cells adapt to sublethal concentrations of chemotherapeutic agents. These limitations underscore the urgent need for advanced delivery systems that can enhance precision by selectively targeting tumor cells while sparing healthy tissues. A summary of current treatment options for colon cancer, along with their respective advantages and disadvantages, is provided in **Table 1**.

**Table 1:** The table summarizing the current therapies for colon cancer, including available treatments with their advantages and disadvantages.

Therapy	Description	Advantages	Disadvantages	References
Surgery	Removal of the tumor and, if necessary, nearby lymph nodes; includes colectomy and laparoscopic surgery.	▪ Potentially curative for early-stage CRC. ▪ Removes localized tumors. ▪ Minimal recurrence risk if complete resection is achieved.	▪ Ineffective for metastatic CRC. ▪ Risks of complications (infection, bleeding, and bowel obstruction).	▪ (55)
Chemotherapy	Systemic use of drugs (e.g., FOLFOX, FOLFIRI) to kill cancer cells; can be neoadjuvant, adjuvant, or palliative.	▪ Effective for shrinking tumors. ▪ Reduces recurrence risk in early-stage disease. ▪ Improves survival in metastatic CRC.	▪ Side effects: nausea, fatigue, hair loss. ▪ Nonspecific action damages healthy cells. ▪ Resistance often develops.	▪ (56)
Radiation Therapy	High-energy radiation to destroy cancer cells, typically for rectal cancer.	▪ Useful for shrinking tumors pre-surgery. ▪ Effective for localized rectal cancer. ▪ Can be combined with chemotherapy for synergy.	▪ Limited application in colon cancer (mostly used for rectal cancer). ▪ Side effects: fatigue, bowel irritation.	▪ (57)
Targeted Therapy	Drugs targeting specific molecular pathways (e.g., VEGF inhibitors like Bevacizumab, EGFR inhibitors like Cetuximab).	▪ Precision targeting of cancer cells. ▪ Reduces damage to healthy tissues. ▪ Extends survival in metastatic CRC.	▪ Not all patients benefit (requires specific biomarkers). ▪ Resistance develops over time. ▪ High cost.	▪ (45)
Immunotherapy	Boosts the immune system to attack cancer cells (e.g., PD-1 inhibitors like Pembrolizumab).	▪ Effective for mismatch repair-deficient (dMMR) or microsatellite instability-high (MSI-H) tumors. ▪ Durable responses in some cases.	▪ Limited to specific subtypes of CRC. ▪ May cause immune-related adverse effects. ▪ Expensive and not widely applicable.	▪ (58)
Neoadjuvant Therapy	Pre-surgical chemotherapy or radiation to shrink tumors.	▪ Facilitates complete tumor resection. ▪ Improves surgical outcomes.	▪ Not suitable for all stages. ▪ Risk of side effects from pre-surgical treatment.	▪ (59)
Adjuvant Therapy	Post-surgical chemotherapy to eradicate residual cancer cells.	▪ Reduces risk of recurrence. ▪ Targets micrometastatic disease.	▪ Adds to treatment burden. ▪ Side effects may impair quality of life.	▪ (59)
Palliative Care	Symptom management and quality-of-life improvement for advanced cancer.	▪ Focuses on patient comfort. ▪ Can incorporate chemotherapy, pain management, and psychosocial support.	▪ Does not aim to cure the disease. ▪ Limited impact on survival.	▪ (60)
Emerging Therapies (e.g., LDEPT)	Ligand-directed therapies to selectively deliver drugs to cancer cells.	▪ High specificity. ▪ Minimizes systemic toxicity. ▪ Promising for drug-resistant cancers.	▪ Still experimental. ▪ Delivery challenges. ▪ High cost and complexity.	▪ (46)

The study by Behr et al. underscores key challenges that continue to limit the clinical translation of targeted enzyme prodrug therapies. Among these, achieving ligand specificity remains critical since the development of high-affinity, tumor-specific ligands are often compromised by off-target binding to normal tissues, thereby reducing selective efficacy. Moreover, the immunogenicity of non-human enzymes used within these systems elicits host immune responses, which can lead to rapid clearance and the production of neutralizing antibodies that impair repeated dosing protocols. Additionally, efficient delivery is further complicated by the heterogeneity of the tumor microenvironment, where variable vascularization and physical barriers impede the uniform distribution of therapeutic enzymes. To overcome these limitations, further research is warranted in the design of humanized ligands and enzymes, the incorporation of immunomodulatory strategies, and the optimization of advanced delivery vehicles—such as nanoparticle-based systems—to enhance the precision and effectiveness of these therapies (61).

Emerging strategies involving novel technologies—such as nanocarrier-based systems and ligand-directed therapies—offer promising solutions to overcome these barriers (62–64). These approaches aim to overcome barriers posed by traditional chemotherapy and improve patient outcomes. Several strategies have been extensively investigated for the selective targeting and delivery of anticancer drugs (63, 65, 66). A key approach involves using functional ligands that bind to receptors overexpressed on cancer cells. Ligands such as folic acid, hyaluronic acid, transferrin, peptides, and antibodies have been studied to develop tumor-specific drug delivery systems (66). Additionally, the use of cell-penetrating peptides or ligands that open tight junctions within tumors such as in the epithelial tissues is actively explored to enhance intracellular drug delivery. These ligands, used alone or in combination, improve target specificity and cellular uptake of anticancer drugs (67).

The following section highlights recent advances in optimizing chemotherapy effectiveness through these novel delivery systems. Specifically, the following section delve deep into the use of functional ligands and their role in targeting receptors overexpressed on cancer cells, marking the next step in the evolution of more effective cancer therapies.

## 7 Strategies for target-specific drug delivery: passive and active targeting in cancer therapies

**Figure 2:**
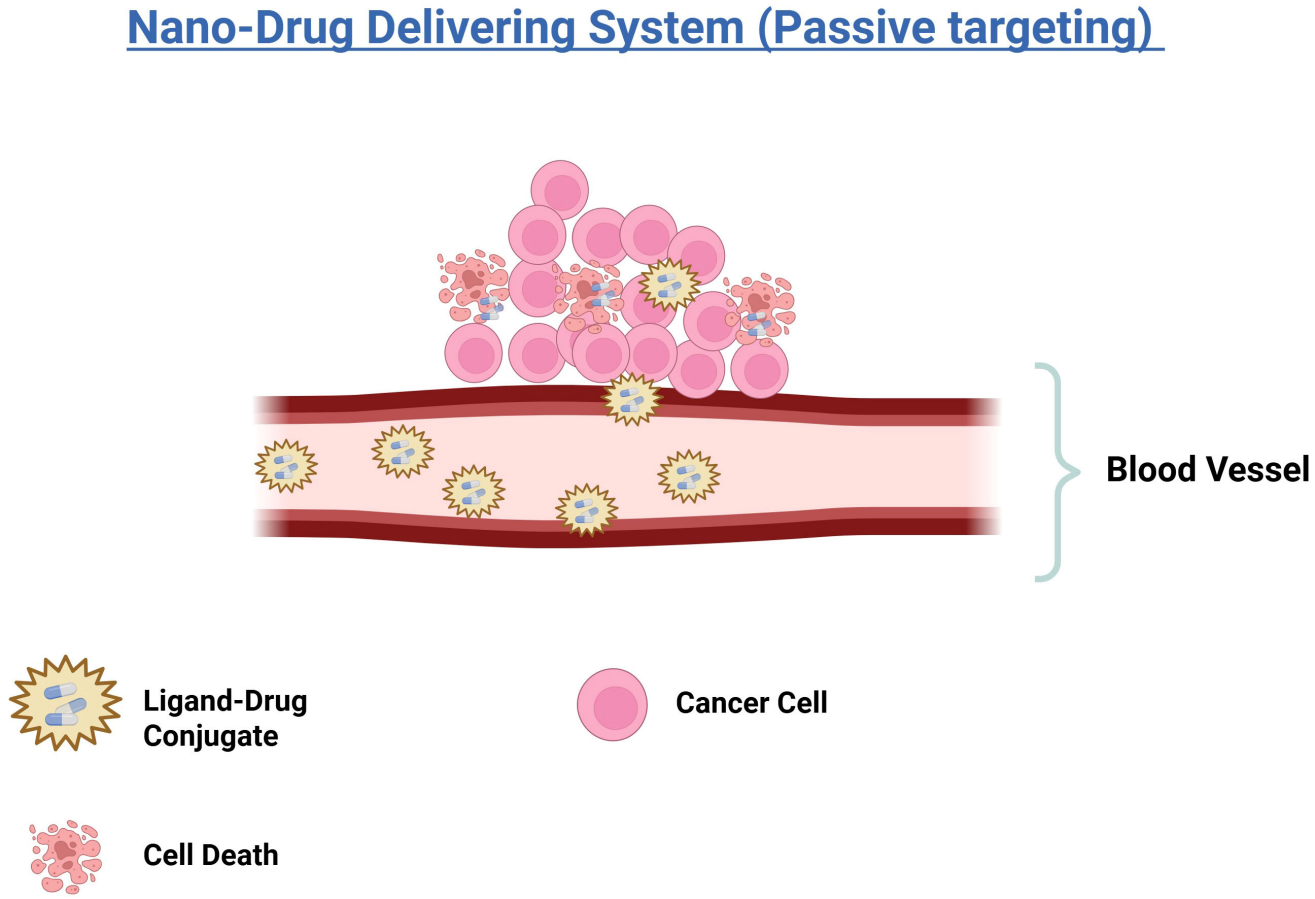
Illustration of a nano-drug delivery system using passive targeting. Ligand-drug conjugates are transported through the bloodstream and accumulate near cancer cells. The accumulation leads to the death of cancer cells, demonstrating the effectiveness of the nano-drug delivery in targeting and eliminating cancerous tissues.

The advancement of drug delivery systems has markedly enhanced the precision of cancer therapies. Passive targeting emerging as a key strategy for the selective delivery of therapeutic agents to tumor sites. This approach harnesses the pharmacological and physicochemical properties of Nano-drug delivery systems (Nano-DDS) (68). The leaky and disorganized blood vessels of tumor tissue, along with inadequate lymphatic drainage, are key features that passive targeting exploits to differentiate tumor vasculature from normal tissues (**Figure 2**) (69). These characteristics facilitate the selective accumulation of Nano-DDS at tumor sites through the enhanced permeability and retention (EPR) effect (68). Nano-DDS with hydrodynamic diameters exceeding the renal clearance threshold remain in circulation longer, which allows them to extravasate through the leaky tumor vasculature (70, 71). Various types of Nano-DDS, including liposomes, polymer-drug conjugates, polymer micelles, PEGylated proteins, plasma proteins, and nanocapsules, utilize the EPR effect for tumor accumulation (68). The effectiveness of passive targeting can be influenced by factors such as the surface charge, hydrophobicity/hydrophilicity, and biocompatibility of the Nano-DDS. Notably, the EPR effect is often more pronounced in small animal tumor models compared to human cancers (72). Recent studies indicate that, on average, only about 0.7% of the injected dose of Nano-DDS reaches tumors through the EPR effect (73), underscoring the debate regarding the extent and reliability of the EPR effect in human tumors (72).

Despite the promising advancements in passive and active targeting strategies, a major limitation in the clinical translation of Nano-DDS is their rapid clearance by the mononuclear phagocyte system (MPS), primarily through macrophage-mediated phagocytosis. This process, known as reticuloendothelial clearance, leads to the preferential accumulation of these nanocarriers in organs rich in phagocytic cells—most notably the liver and spleen (74, 75). As a result, a significant portion of the administered Nano-DDS dose fails to reach the tumor site, thereby reducing therapeutic efficacy and increasing off-target effects. To address this challenge, surface modifications such as polyethylene glycol (PEG)ylation and silica coating have been employed to enhance the systemic stability and circulation time of Nano-DDS. These modifications reduce opsonization and subsequent recognition by macrophages, allowing for improved bioavailability and tumor-specific delivery (76, 77). PEGylation, in particular, has become a widely adopted strategy to create ‘stealth’ nanoparticles, minimizing immune clearance and enhancing therapeutic outcomes.

**Figure 3:**
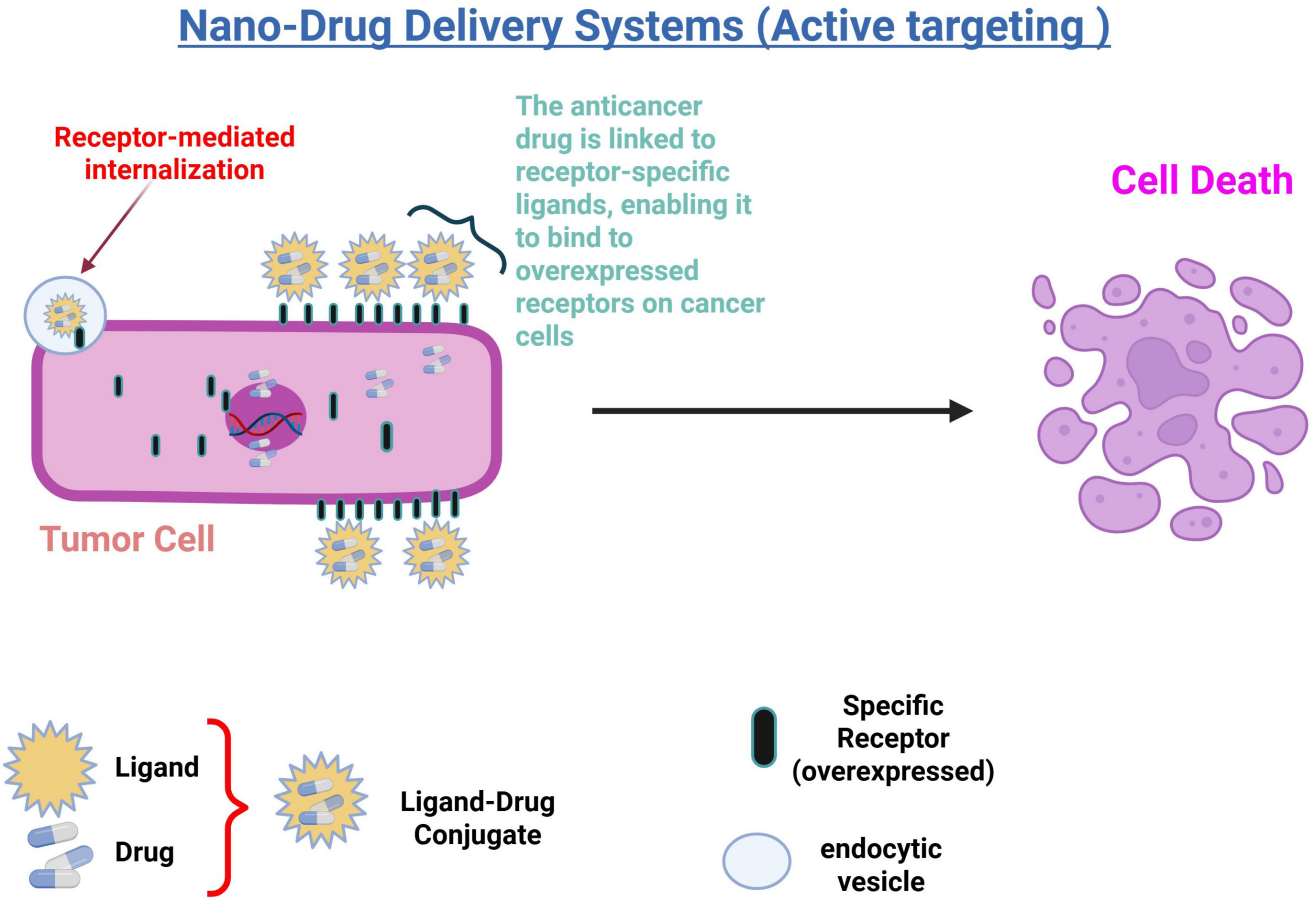
Illustration of nano-drug delivery systems using active targeting: In this process, anticancer drugs are linked to receptor-specific ligands, forming a ligand-drug conjugate that binds to overexpressed receptors on cancer cells. This interaction triggers receptor-mediated internalization, allowing the drug to enter the cell via endocytic vesicles. The targeted delivery enhances drug accumulation in tumor cells, leading to increased efficacy and subsequent cell death.

To further enhance the precision and effectiveness of drug delivery systems, active targeting strategies have been developed. These strategies aim to increase the specificity of Nano system-delivered drugs for their target sites, thus improving therapeutic outcomes. Active targeting is achieved through various methods, such as ligand conjugation and adsorption, which direct the Nano systems more precisely to the tumor (78). Active targeting improves the effectiveness of Nano system-delivered drugs by increasing specificity for the target site. This is achieved by either conjugating specific ligands to overexpressed tumor receptors or through adsorption. The ligand facilitates receptor-mediated endocytosis, enhancing drug uptake into tumor cells, as shown in (**Figure 3**). Studies have shown that functionalizing ligands on Nano systems significantly boosts therapeutic efficacy compared to native drugs or passively targeted systems (50, 69).

## 8 Functionalization of delivery vehicles for targeted tumor delivery

Effective tumor targeting relies not only on the selection of appropriate ligands but also on how these ligands are attached to delivery systems such as nanoparticles, liposomes, and micelles. Common strategies for ligand functionalization include chemical conjugation, electrostatic interactions, and hydrophobic insertions.

Chemical conjugation, such as amide or thiol–maleimide linkages, enables stable covalent bonding between ligands and nanocarrier surfaces, often used for attaching antibodies and peptides (79). Electrostatic interactions offer a simpler, non-covalent approach by exploiting charge differences between ligands and carriers, though these are generally less stable in physiological environments. Hydrophobic insertions, frequently used in lipid-based systems, allow lipophilic ligands to integrate into the lipid core or bilayer of the carrier (80).

These functionalization strategies directly impact the stability, circulation time, and targeting efficiency of the Nano-DDS, and their careful optimization is key to improving therapeutic outcomes.

## 9 Peptide power: targeting cancer with precision

In recent years, peptide ligands have emerged as powerful tools in targeted drug delivery, owing to their specificity, biocompatibility, and ability to engage in precise interactions with cell surface receptors. Peptide ligands, which are short chains of amino acids, can be engineered to recognize and bind to specific targets, such as overexpressed receptors on cancer cells. This ability to selectively home in on disease sites offers significant advantages in therapeutic applications, including reduced off-target effects and enhanced drug efficacy (81, 82). The effectiveness of peptide ligands in drug delivery is enhanced by their favorable hydrodynamic properties, which promote efficient distribution and retention within biological fluids. By optimizing these properties, researchers can increase the stability, circulation time, and tissue penetration of peptide-based therapeutics. This makes peptide ligands an increasingly appealing option for developing innovative drug delivery systems that improve treatment accuracy and patient outcomes. Moreover, peptide ligands offer several advantages, including high specificity for their targets, ease of synthesis, and low immunogenicity, making them particularly well-suited for targeted drug delivery applications (82). Often referred to as tumor-targeting or cell-targeting peptides (82), these peptides are identified through bio-inspired techniques or large-scale peptide library screenings, such as phage display and chemical libraries (81, 83). They vary widely in origin, structure, and application, offering extensive resources for precise drug delivery (84, 85).

Several peptide motifs have been identified that specifically target cancer cells, including NGR and RGD peptides. While natural proteins and peptides can act as effective ligands for cancer-related receptors, their direct use in targeted delivery is often limited by issues like poor biocompatibility and high toxicity (86, 87). However, structure-based optimization can produce biomimetic peptides that overcome these limitations, offering benefits such as enhanced stability and specificity. For example, octreotide, a synthetic analogue of somatostatin, has been utilized for targeted delivery of various therapeutic agents (87). Unlike structure-based biomimetic design, phage display enables the discovery of peptide ligands without prior knowledge of their binding properties, leading to the rapid identification of novel peptides (87). Over the past decades, this method has uncovered several peptide motifs that specifically target cancer cells, such as NGR (asparagine-glycine-arginine) and RGD (arginine-glycine-aspartic acid) (88). The RGD peptide targets integrins, which are heterodimeric glycoproteins overexpressed in the endothelial cells of tumor vasculature (89). Integrins, consisting of α and β subunits, show specificity and differential affinity in binding their ligands. The RGD peptide specifically binds to αvβ3 and αvβ5 integrins, facilitating its accumulation in tumor vasculature (90). The findings demonstrated that nanoparticles decorated with RGD peptides significantly inhibited cancer cell proliferation and exhibited a lower IC50 compared to nanoparticles lacking RGD peptides. This indicates that RGD peptides have strong potential as effective ligands for targeting tumors (90). A broader overview of novel ligand-targeted drug delivery strategies developed for colorectal cancer is summarized in **Table 2**.

**Table 2:** Overview of Novel Ligand-Targeted Drug Delivery Strategies in Colorectal Cancer.

Novel Ligand	Drug Delivery System (DDS)	Study Description	Major Outcomes	Reference
Anisamide	Lipidic Core Nanocapsules	Development of thymoquinone-loaded nanocapsules targeted with anisamide to target sigma receptors overexpressed in CRC cells.	Enhanced cytotoxicity against HT-29 CRC cells overexpressing sigma receptors compared to non-targeted nanocapsules.	(91)
γδ T Cells	Immunotherapy Approach	Investigation of γδ T cells’ role in CRC, focusing on their cytotoxic activity through granzyme B and perforin release.	γδ T cells exhibit potent cytotoxic activity against CRC cells, suggesting potential for immunotherapeutic strategies.	(92)
Folic Acid	Folic Acid-Conjugated Nanogels	Use of folic acid-conjugated nanogels loaded with chemotherapeutic agents to target folate receptor-overexpressing CRC cells.	Significant decrease in tumor growth in mouse models, demonstrating effective site-specific delivery.	(74)
Benzimidazole Antihelminthics	Small Molecule Therapeutics	Evaluation of benzimidazole derivatives for their anticancer activity in CRC models.	Induced apoptosis and cell cycle arrest in CRC cells with minimal cytotoxicity to normal cells.	(93)
Exosomal Anthocyanidins (ExoAnthos)	Exosome-Based Delivery	Encapsulation of berry-derived anthocyanidins in bovine milk-derived exosomes for targeted delivery to CRC cells.	Reduced tumor mass and decreased tumor proliferation in mice models, indicating potential for CRC prevention and therapy.	(94)

This table summarizes recent advancements in ligand-targeted drug delivery systems (DDS) developed for colorectal cancer treatment. It highlights the variety of ligands explored, the innovative delivery platforms used, and the therapeutic outcomes achieved, including tumor reduction and enhanced cytotoxicity.

## 10 Optimizing chemotherapy through ligand-based targeting: a new era in oncology

By combining the specificity of ligand-directed delivery with the potent cytotoxic effects of chemotherapy, LDEPT offers a promising approach to enhance therapeutic efficacy and minimize systemic toxicity. The epidermal growth factor receptor (EGFR) plays a critical role in the development and progression of colorectal cancer (CRC). While anti-EGFR therapies have significantly improved patient outcomes, their effectiveness is often limited by factors such as drug resistance and adverse side effects. To overcome these challenges, innovative strategies are needed to enhance the targeted delivery of therapeutic agents to cancer cells (95).

EGFR, a member of the ERBB/HER family, is overexpressed in 25%-77% of CRC cases (96). Anti-EGFR therapies have been most effective in patients with wild-type (WT) RAS mutations, but they are largely ineffective in those with BRAF mutations, which occur independently of RAS mutations. As a result, current clinical guidelines recommend anti-EGFR therapy primarily for patients with both BRAF-WT and RAS-WT tumors (97, 98). Moreover, EGFR expression is more pronounced in left-sided CRCs compared to right-sided tumors, with patients having left-sided tumors showing a more favorable response to anti-EGFR therapy (99). However, resistance to anti-EGFR therapy typically develops within 3-12 months, primarily due to mutations in downstream signaling pathways such as RAS/RAF/MEK, PI3K/AKT/mTOR, and the activation of compensatory pathways, including ERBB2 and MET (100–102). A summary of currently FDA-approved targeted therapies for metastatic colorectal cancer is presented in **Table 3**. To overcome these limitations and address the emerging resistance to EGFR-targeted therapies, LDEPT offers a promising strategy.

**Table 3:** Summary of FDA-approved targeted therapies for metastatic colorectal cancer.

Target/Pathway	Drug(s)	Mechanism of Action	Key Findings/Trials	FDA Approval/Notes	Reference(s)
EGFR	Cetuximab	Chimeric IgG mAb blocks EGFR → inhibits downstream signaling	BOND trial: 22.9% ORR; CRYSTAL III: ↓ progression by 15% (HR = 0.85; P = 0.048)	FDA approved 2004; KRAS WT only; acneiform rash, electrolyte imbalance	(97, 103–106)
	Panitumumab	Fully human mAb blocks EGFR, induces apoptosis, inhibits angiogenesis & ADCC	PRIME trial: ↑ PFS in KRAS WT (9.6 vs 8 months); BSC study: ↑ OS (HR = 0.72), ↑ PFS (HR = 0.45)	Similar efficacy to cetuximab; fewer hypersensitivity issues; side preference may vary	(107–111)
VEGF/VEGFR	Bevacizumab	Anti-VEGF-A mAb → inhibits angiogenesis	Phase III: IFL + bevacizumab ↑ OS (20.3 vs 15.6 months), ↑ PFS (10.6 vs 6.2 months)	FDA approved 2004; more adverse events than anti-EGFR agents	(112–115)
	Fruquintinib	Oral TKI targeting VEGFR1–3	FRESCO-2: ↑ OS (7.4 vs 4.8 months), HR = 0.66 (P < 0.0001); common: hypotension, asthenia	FDA approved 2023 for mCRC after standard therapy	(116, 117)
	Aflibercept	VEGFR1/2-Fc fusion protein → decoy receptor	VELOUR trial: with FOLFIRI ↑ OS (HR = 0.857; P = 0.0032)	FDA approved 2012; common: HTN, thrombosis, diarrhea, neutropenia	(118, 119)
	Ramucirumab	Fully human mAb targets VEGFR2	RAISE trial: with FOLFIRI ↑ OS (HR = 0.844; P = 0.0219)	Second-line mCRC; common AEs: neutropenia, HTN, diarrhea, fatigue	(120)
	Regorafenib	Oral multitarget TKI (VEGFRs, PDGFR, FGFR, RET, etc.)	CORRECT trial: ↑ OS (HR = 0.77; P = 0.0052); superior to aflibercept, ramucirumab in PFS	Third-line agent; used after standard treatments	(121, 122)
BRAF-V600E	Encorafenib + Cetuximab (± Binimetinib)	BRAF inhibitor + anti-EGFR (± MEK inhibition)	BEACON trial: ↑ OS (9.3 vs 5.9 months; HR = 0.60) in mCRC with BRAF-V600E mutation	FDA approved 2020; ASCO-recommended for progressed BRAF-V600E mCRC	(106, 123, 124)
HER2 (ErbB2)	Trastuzumab + Tucatinib	HER2 mAb + HER2-selective oral TKI	MOUNTAINEER trial: ORR = 38.2%; duration of response = 12.4 months	Accelerated FDA approval Jan 2023 for HER2+ mCRC post chemotherapy	(125–127)

↑, Increase or improvement; ↓, Decrease or reduction.

Ligand-Directed Enzyme Prodrug Therapy (LDEPT) presents a promising solution to address the limitations of conventional chemotherapy. This approach uses specific ligands to deliver enzymes directly and specifically to tumor cells, enabling the precise activation of prodrugs at the tumor site. This localized activation maximizes therapeutic efficacy while minimizing systemic toxicity. LDEPT has the potential to revolutionize CRC treatment, particularly for patients with advanced disease or those who have developed resistance to traditional therapies (128).

**Figure 4:**
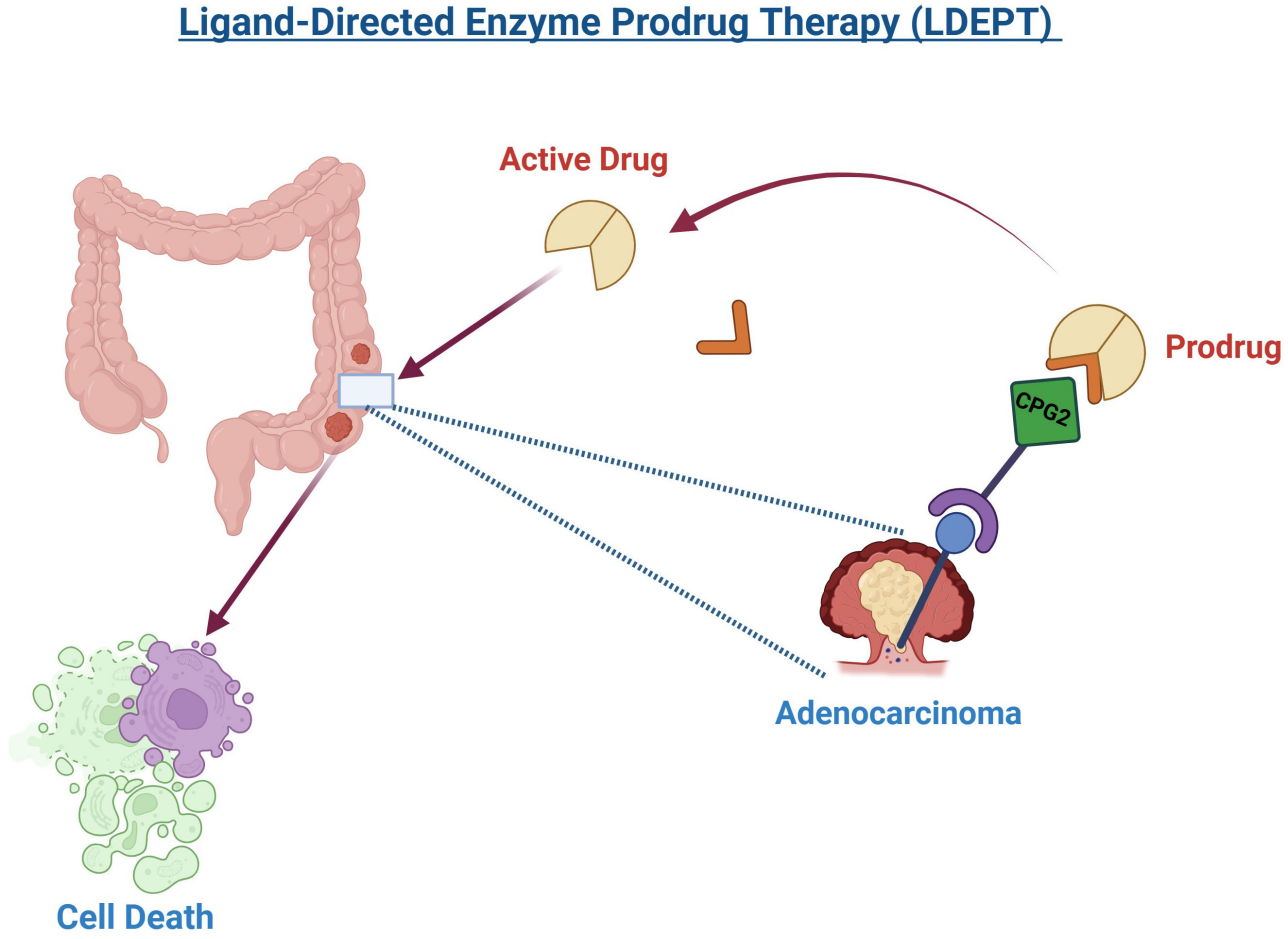
Ligand-Directed Enzyme Prodrug Therapy (LDEPT) utilizes a ligand, such as a peptide, to guide an enzyme to a tumor site, specifically adenocarcinoma in this illustration. Upon reaching the tumor, the enzyme, carboxypeptidase G2 (CPG2), activates a prodrug, converting it into its cytotoxic form. This targeted approach leads to the destruction of cancer cells (cell death) while minimizing damage to surrounding healthy tissues. The process exemplifies a promising method in precision cancer therapy.

Given these limitations and the emergence of resistance, there is an increasing need to explore alternative treatment strategies. LDEPT offers an innovative targeted approach by using a ligand to selectively deliver an enzyme to cancer cells, where it activates a prodrug specifically at the tumor site (**Figure 4**). This method has the potential to enhance therapeutic efficacy while minimizing off-target effects and overcoming resistance mechanisms. Integrating LDEPT could thus provide a more effective and personalized treatment option for CRC patients, especially those with resistant or difficult-to-treat tumors (129). Various enzymes have been investigated for use in LDEPT, with carboxypeptidase G2 (CPG2) emerging as a key enzyme for clinical application (130). CPG2 is an exopeptidase that converts synthetic non-toxic “benzoic mustard prodrugs” into cytotoxic agents, making it highly suitable for targeted cancer therapy. In addition to its role in cancer treatment, CPG2 is also used to detoxify patients who have accidentally overdosed on methotrexate (MTX), a commonly used chemotherapeutic agent (131, 132).

A promising study has demonstrated the potential of Ligand-Directed Enzyme Prodrug Therapy (LDEPT) by engineering fusion proteins that exploit ligand–enzyme specificity for targeted cancer therapy. In this approach, the enzyme carboxypeptidase G2 (CPG2) was genetically fused to the cyclic peptide CNGRC, which selectively binds to aminopeptidase N (APN)—a tumor-associated antigen overexpressed in various solid malignancies. Two constructs were designed: one with a single CNGRC motif (X-CPG2) and another with CNGRC peptides at both termini (X-CPG2-X). These ligands facilitated specific binding to APN-expressing tumor cells, enhancing targeted localization of the enzyme. The double-fused protein (X-CPG2-X) not only showed stronger binding affinity but also increased catalytic efficiency, likely due to favorable conformational changes in the enzyme structure. Functionally, these fusion proteins successfully activated the prodrug ZD2767P into a potent cytotoxic agent, causing selective killing of APN-high cancer cells *in vitro*, while sparing APN-low cells. Moreover, the constructs were effective in reducing methotrexate toxicity by enzymatically degrading the drug, demonstrating dual therapeutic benefit. Importantly, these fusion proteins showed reduced immunogenicity in T-cell assays and maintained enzymatic activity for over 14 days in serum, underscoring their clinical promise. Together, these findings represent a successful preclinical example of LDEPT that integrates specific ligand–enzyme interactions with efficient prodrug activation, offering a compelling strategy for tumor-selective chemotherapy (133).

The current landscape of prodrug activation strategies in cancer treatment includes several promising approaches that aim to improve specificity and reduce off-target toxicity. One such strategy involves using upconverting nanoparticles (UC) conjugated with cytosine deaminase (CD), which activates prodrugs at tumor sites under near-infrared (NIR) light. This method demonstrated significant tumor targeting and localized drug activation, resulting in extended survival in animal models (134). In contrast, thymidine phosphorylase (TP) gene therapy aims to enhance the sensitivity of tumor cells to prodrugs such as 5-fluorouracil (5-FU) and 5′-deoxy-5-fluorouridine (5′-DFUR) by transfecting cancer cells with TP. This approach has shown increased cytotoxicity and a bystander effect, improving treatment efficacy (135). Another innovative approach involves the use of magnetic nanoparticles conjugated with 2′-deoxyribosyltransferase (PDT-MIONP), which selectively activates purine-based prodrugs in tumor cells, demonstrating a high degree of specificity and reduced off-target effects (136). Additionally, CPG2 as a prodrug activator offers significant advantages, particularly in its ability to activate methotrexate-based prodrugs. CPG2 enzyme has been shown to enhance selective cytotoxicity in tumor cells when conjugated with targeting ligands, offering localized activation of prodrugs with minimal systemic toxicity. Compared to other enzyme candidates, CPG2 has shown advantages in terms of stability, reduced immunogenicity, and higher enzyme activity, making it a promising option for targeted cancer therapies. This specificity reduces the risk of collateral damage to healthy tissues, a major issue with traditional chemotherapy (133).

These strategies highlight the potential for tailored, localized prodrug activation that could significantly improve the precision and efficacy of cancer treatments. Below is a comparative summary of these strategies (**Table 4**).

**Table 4:** Comparative overview of CRC treatment strategies.

Strategy	Mechanism	Prodrug Activated	Target	Key Results	Advantages
Upconverting Nanoparticles (UC) and Cytosine Deaminase (CD) Conjugates	Photo-cross-linkable conjugate activated by NIR light to bind EGFRs on tumor cells	5-fluorocytosine (5-FC) converted to 5-fluorouracil (5-FU)	Tumors overexpressing EGFR (e.g., Caco-2 cells)	Increased tumor accumulation (5-fold) and slower tumor growth (2-fold), survival increase from 28 to 35 days	NIR-controlled targeting, extended survival, localized prodrug activation
Thymidine Phosphorylase (TP) in Gene Therapy	Transfection of cancer cells with TP to increase sensitivity to pyrimidine prodrugs	5-fluorouracil (5-FU) and 5′-deoxy-5-fluorouridine (5′-DFUR)	TP-transfected cancer cells (e.g., LS174T)	80-fold decrease in IC50 for 5-FU, 40-fold decrease for 5′-DFUR, bystander effect	Enhanced sensitivity to prodrugs, bystander effect, gene therapy approach
Magnetic Nanoparticle-Conjugated 2′-Deoxyribosyltransferase (PDT-MIONP)	Magnetic nanoparticles covalently attached to Leishmania mexicana 2′-deoxyribosyltransferase (PDT)	2-fluoro-2′-deoxyadenosine (dFAdo)	Tumor cells (e.g., HeLa)	11% viability in tumor cells (HeLa), higher intracellular uptake in tumor cells	High specificity for tumor cells, reduced off-target effects, magnetic targeting
CPG2 Prodrug Activation	Enzyme-based activation of methotrexate prodrugs by CPG2	Methotrexate (MTX) and its derivatives	Tumor cells, especially those with a suitable CPG2-targeting ligand	Increased selectivity and cytotoxicity for tumor cells	High specificity, reduced off-target toxicity, minimal systemic effects

## 11 Adverse effect profile of targeted cancer therapies

Targeted therapies have transformed cancer treatment by improving selectivity towards malignant cells, yet they exhibit a unique spectrum of adverse effects. For instance, agents such as EGFR inhibitors commonly induce dermatologic toxicities—including rash, dry skin, and paronychia—due to interference with EGFR signaling in normal skin cells. In addition, gastrointestinal disturbances (e.g., diarrhea, nausea, vomiting, mucositis) are frequently reported, reflecting the impact of these drugs on normal epithelial function. Cardiovascular effects, such as hypertension, proteinuria, and an increased risk of thromboembolic events, are particularly associated with agents that disrupt vascular endothelial growth factor (VEGF) signaling. Moreover, alterations in liver function and endocrine abnormalities, such as hypothyroidism, can occur secondary to direct toxicity or immune-mediated mechanisms. Although these adverse effects are generally more manageable than those associated with conventional cytotoxic chemotherapies, they necessitate vigilant monitoring and tailored supportive care to maintain optimal therapeutic outcome (137).

## 12 Enhancing cancer treatment: ADEPT and LDEPT as targeted prodrug therapy innovations

Targeted cancer therapies have transformed the field of oncology by enabling the selective elimination of tumor cells while minimizing damage to surrounding healthy tissues. One such promising approach is Methotrexate can cause severe adverse effects, particularly renal dysfunction and, in extreme cases, renal failure. The rapid clearance of methotrexate is therefore essential, and CPG2 facilitates this process by hydrolyzing the carboxyl terminal glutamate moiety of methotrexate, producing the safer byproducts glutamic acid and 2,4-diamino-N10-methylpteroic acid (DAMPA) (138). Due to its dual role in cancer therapy and methotrexate detoxification, CPG2 is regarded as a valuable enzyme in oncological research. A significant advantage of ADEPT lies in its ability to target not only antigen-expressing cancer cells but also nearby tumor cells through the bystander effect. In this process, cytotoxic agents generated from the activated prodrug diffuse into the surrounding environment, thereby extending the therapeutic effect to neighboring cancer cells that may not express the target antigen. This enhances the overall efficacy of ADEPT. Clinical trials have shown encouraging tumor responses using this strategy (139). However, two major challenges may limit its effectiveness: (i) the restricted availability of suitable tumor-specific markers for antibody targeting, and (ii) the immunogenicity of non-human enzymes, which can result in significant systemic toxicity when repeatedly administered in clinical settings (140).

Enzyme prodrug systems for cancer gene therapy face significant challenges due to immunogenicity, particularly when using enzymes of non‐human origin. For example, CPG2, a bacterial enzyme used in ADEPT, is highly immunogenic; its non‐human origin triggers robust host immune responses, leading to the rapid development of anti-CPG2 antibodies that accelerate its clearance and neutralize its activity, thereby compromising therapeutic efficacy and complicating repeat dosing (141). Similarly, although rabbit carboxylesterase efficiently converts irinotecan to SN-38, its immunogenicity has necessitated the engineering of humanized variants (e.g., hCE1m6) to reduce immune activation while preserving function. In addition, the inherent immunogenicity of gene delivery vectors further exacerbates these challenges, making strategies such as enzyme pegylation and protein engineering crucial for improving the safety and effectiveness of enzyme prodrug systems (142).

Ligand-Directed Enzyme Prodrug Therapy (LDEPT) is an advanced targeted cancer treatment approach where a protein or peptide ligand directs an enzyme to the tumor site. There, the enzyme converts a prodrug into its active cytotoxic form, leading to localized cancer cell death. Unlike Antibody-Directed Enzyme Prodrug Therapy (ADEPT), which uses larger antibodies or antibody fragments covalently attached to the enzyme, LDEPT involves smaller fusion proteins that offer several advantages (143).

One major advantage of LDEPT is the smaller size of the fusion proteins, which makes them relatively inexpensive to produce. Manufacturing smaller proteins is less complex and more cost-effective compared to ADEPT systems that require larger antibody-enzyme conjugates (131). Furthermore, these smaller fusion proteins exhibit fewer solubility issues, which is an important factor in their distribution and activity within the body. Improved solubility allows for better tumor penetration and enhanced bioavailability, increasing the efficacy of the therapy (144). Another advantage of smaller fusion proteins is their ability to diffuse more easily through the tumor microenvironment, ensuring better access to cancer cells (145).

Despite these benefits, LDEPT does face some challenges. One significant disadvantage is the limited specificity of the smaller protein or peptide ligands compared to antibodies used in ADEPT. While antibodies can achieve a high degree of selectivity for tumor-associated antigens, the ligands in LDEPT may exhibit lower affinity and specificity, which increases the risk of off-target effects and unintentional damage to healthy tissues (146). Moreover, smaller fusion proteins often have shorter circulatory half-lives, potentially requiring more frequent administration to maintain therapeutic levels at the tumor site (133). The risk of systemic toxicity also exists, especially if the prodrug is prematurely activated in non-tumor tissues. Below is a comparative table that summarizes the key differences between ADEPT and LDEPT, highlighting their distinct targeting strategies, pharmacokinetic profiles, and inherent challenges (**Table 5**).

**Table 5:** Comparative overview of ADEPT vs. LDEPT approaches.

Parameter	ADEPT	LDEPT	References
Targeting Agent	Utilizes monoclonal antibodies, which provide high specificity for tumor-associated antigens	Employs smaller ligands such as peptides, aptamers, or small proteins that can be tailored for tumor targeting	(131, 139) vs (133, 147)
Molecular Size and Tumor Penetration	Large antibodies can hinder deep tumor penetration and result in prolonged circulation times, potentially leading to increased off-target exposure and toxicity	Smaller ligands enable improved tumor penetration and rapid clearance from non-target tissues, reducing systemic exposure and off-target effects	(131, 139) vs (133, 147).
Immunogenicity	Higher risk of immunogenic responses due to the inherent properties and size of antibodies	Reduced immunogenicity risk owing to the smaller, more easily humanized ligands	(131, 139) vs (133, 147).
Flexibility and Cost	High specificity but often entails complex engineering and higher production costs	Greater engineering flexibility and typically more cost-effective production methods	(131, 139) vs (133, 147).
Overall Challenges	Limited by issues such as suboptimal tumor penetration, prolonged circulation, and off-target toxicity; challenges are further compounded by the tumor microenvironment	Requires optimization of ligand specificity and delivery; similar challenges exist with respect to heterogeneous tumor microenvironments, but the smaller size may help mitigate some limitations	

In summary, while LDEPT offers advantages in terms of cost, solubility, and tumor penetration, careful consideration of ligand specificity and pharmacokinetics is essential for optimizing its clinical efficacy and minimizing side effects.

## 13 Conclusion

Colorectal cancer remains a significant challenge in oncology, particularly in advanced stages where conventional therapies often fall short. Standard treatments such as surgery, chemotherapy, and radiation therapy, though effective in early-stage disease, face numerous limitations in metastatic or recurrent CRC, including the development of drug resistance, severe toxicity, and limited specificity toward cancer cells (37, 148). As a result, there is a pressing need for more innovative and targeted therapeutic approaches that can overcome these barriers while improving patient survival and quality of life (22).

Ligand-Directed Enzyme Prodrug Therapy (LDEPT) emerges as a promising solution to these challenges. By leveraging the ability of ligand-enzyme complexes to selectively bind to cancer cells, LDEPT enables highly specific drug activation at tumor sites, reducing off-target effects and minimizing damage to healthy tissues (131, 149). This targeted approach not only improves the efficacy of treatment but also addresses key issues like drug resistance and systemic toxicity that are common in conventional chemotherapy (51, 133). The use of smaller fusion proteins and ligands with high affinity for cancer cells enhances tumor penetration, making LDEPT a potentially superior option in cases where other therapies are ineffective (139).

Preclinical studies have demonstrated LDEPT’s potential in achieving greater therapeutic precision, while early-phase clinical trials have shown encouraging results, suggesting that this approach could revolutionize CRC treatment, particularly for patients with advanced disease (138, 147). However, challenges remain, including the optimization of ligand specificity, drug delivery methods, and pharmacokinetics, as well as addressing potential immune responses and ensuring sustained enzyme activity at tumor sites (40, 150). These hurdles must be carefully navigated through further research, technological innovations, and clinical validation (151).

The future of LDEPT lies in its potential integration with other therapies, such as immunotherapy or conventional chemotherapy, to create more comprehensive and personalized treatment regimens (46). One promising immuno‐chemotherapy approach involves the combination of platinum‐based chemotherapeutic agents with immune checkpoint inhibitors. In this regimen, the cytotoxic effects of platinum compounds induce immunogenic cell death, resulting in the release of tumor-associated antigens that prime and activate the host immune response. Concurrent treatment with PD-1/PD-L1 inhibitors mitigates the tumor’s immune evasion mechanisms, thereby enhancing T-cell activation and promoting a robust antitumor effect. This synergistic strategy effectively integrates the direct tumor-killing ability of chemotherapy with the immunomodulatory benefits of immunotherapy, and it helps to reinvigorate T-cell responses within the tumor microenvironment, offering the potential for improved clinical outcomes in colorectal cancer (152, 153). Advances in genetic profiling and biomarker identification will likely play a crucial role in tailoring LDEPT to individual patients, further enhancing its effectiveness (36, 73). Moreover, innovations in drug delivery systems, such as nanoparticle carriers or other nanotechnology-based platforms, could improve the precision and bioavailability of the therapeutic agents used in LDEPT (64, 146).

LDEPT holds considerable promise as a next-generation treatment for CRC, offering the potential to significantly improve outcomes for patients, particularly those with drug-resistant or advanced disease. Continued research, clinical trials, and technological advancements are essential to fully realize the therapeutic potential of LDEPT, making it a key player in the future of personalized cancer therapy (154). With its ability to enhance specificity, reduce toxicity, and overcome treatment resistance, LDEPT may play a pivotal role in transforming the landscape of CRC management, ultimately contributing to better survival rates and improved quality of life for patients (96).

In conclusion, while this review demonstrates the promising potential of LDEPT in transforming colorectal cancer treatment, several critical steps remain for its clinical translation. Future research should focus on optimizing drug delivery systems, refining dosing regimens, and conducting rigorous clinical trials to validate both safety and efficacy. Securing funding from national health agencies, research foundations, and promoting industry collaborations are essential to drive these advancements. Moreover, ongoing research efforts—including studies on synergistic combination therapies—will further enhance our understanding and application of targeted enzyme prodrug therapies. Finally, ethical considerations such as ensuring patient safety, obtaining informed consent, and promoting equitable access to these advanced treatments are paramount as we transition LDEPT from experimental studies to routine clinical practice.
